# Investigating Endothelial Activation and Oxidative Stress in relation to Glycaemic Control in a Multiethnic Population

**DOI:** 10.1155/2012/386041

**Published:** 2012-12-06

**Authors:** E. M. Brady, D. R. Webb, D. H. Morris, K. Khunti, D. S. C. Talbot, N. Sattar, M. J. Davies

**Affiliations:** ^1^Department of Diabetes Research, University Hospitals of Leicester, NHS Trust, Leicester LE5 4PW, UK; ^2^Department of Cardiovascular Sciences, University of Leicester, Leicester LE3 9QP, UK; ^3^Department of Health Sciences, University of Leicester, Leicester LE1 6TP, UK; ^4^Unilever Discover, Colworth Science Park, Sharnbrook MK44 ILQ, UK; ^5^BHF Institute for Cardiovascular and Medical Sciences, University of Glasgow, Glasgow G12 8QQ, UK

## Abstract

*Aim*. An exploration of ethnic differences in measures of oxidative stress and endothelial activation in relation to known cardiovascular risk factors within South Asians (SA) and White Europeans (WE) residing in the UK. *Methods*. 202 participants within a UK multiethnic population provided biomedical and anthropometric data. Human urinary 2,3-dinor-8-iso-prostaglandin-F1**α** and plasma ICAM-1 were quantified as measures of oxidative stress and endothelial activation, respectively. *Results*. 2,3-Dinor-8-iso-prostaglandin-F1**α** levels were significantly higher in the SA group compared to WE group (10.36 (95% CI: 9.09, 11.79) versus 8.46 (7.71, 9.29), *P* = 0.021) after adjustment for age, gender, smoking status, body weight, HbA1c, and medication. Oxidative stress was positively associated with HbA1c (*β* = 1.08, 95% CI:1.02, 1.14, *P* = 0.009), fasting *(β* = 1.06, 95% CI: 1.02, 1.10, *P* = 0.002), and 2 hr glucose (*β* = 1.02, 95% CI: 1.00, 1.04, *P* = 0.052). In each adjusted model, SA continued to have elevated levels of oxidative stress compared to WE. ICAM-1 levels were significantly higher in the composite IGR group compared to the normoglycaemic group (*P* < 0.001). No ethnic differences in ICAM-1 were observed. *Conclusion*. These results suggest that SA are more susceptible to the detrimental effects of hyperglycaemia-induced oxidative stress at lower blood glucose thresholds than WE. Further research into the potential mechanisms involved is warranted.

## 1. Introduction

Recent major advances in the treatment of cardiovascular disease have so far failed to rectify recognised ethnic health inequalities in the Northern Hemisphere. For example, first and second generation UK South Asians (people tracing ancestry to India, Pakistan, Bangladesh, or Sri Lanka are termed South Asians) have disproportionately higher rates of coronary heart disease and are more likely to die earlier from cardiovascular causes than their White European counterparts [1]. Whilst an increased prevalence of diabetes within this South Asian diaspora is a well-recognised contributory factor, this and other established risk factors appear not to entirely account for continued discrepancies in rates of cardiovascular morbidity [2–5]. It is plausible that an alternative, unexplained pathophysiology drives premature arterial thromboembolic disease within this ethnic group.

Oxidative stress is probably an important determinant of conditions sharing proinflammatory aetiology, including diabetes mellitus [6, 7], hypertension [8], and atherosclerosis [9]. Failure of endogenous antioxidant defences to control deleterious reactive oxygen species (ROS) leads to protein and lipid peroxidation, the stable endproducts of which serve as increasingly reliable markers of excess ROS defining oxidative stress [10]. F_2_-isoprostane is a widely researched molecule which may have utility as a biomarker in obesity [11], hypercholesterolemia [12], type 2 diabetes mellitus [13], cardiovascular disease [14], and coronary heart disease [15, 16]. Whether South Asians are particularly susceptible to glucose-related oxidative stress or incur its detrimental consequences at lower levels of exposure is unknown.

An intact healthy endothelium is essential for normal vascular and haemodynamic function. Two established surrogates of endothelial pathology, flow-mediated dilation [17], and circulating soluble adhesion molecule (ICAM-1) [18–22] are highly linked to established cardiovascular risk factors [17, 18, 20–22] and have recently been reported to be strong predictors of T2DM [23].

There are few studies exploring the relationship between hyperglycaemia, oxidative stress, (as measured by  F_2_-isoprostanes), and markers of endothelial activation within high-risk ethnic minority groups. Particularly, interplay between postprandial hyperglycaemia, oxidative stress, and endothelial activation may be an important determinant of early arterial wall atherosclerosis within this population.

The purpose of this study was to explore ethnic differences in measures of oxidative stress and endothelial activation and relate these to known cardiovascular risk factors within a well-characterised population cohort. We hypothesise that oxidative stress and endothelial activation will be more prevalent in South Asians with impaired glucose tolerance compared to well-matched white Europeans and will relate to postprandial glucose within this group.

## 2. Methods

### 2.1. Subjects

Subjects were recruited from a community-based screening programme for type 2 diabetes (ADDITION-Leicester), the design of which is described elsewhere [24]. Briefly 1689 South Asians aged 25–75 yrs and 5060 white Europeans aged 40–75 yrs were screened with a standard 75 g-oral glucose tolerance test (OGTT). Additional blood was taken for determination of a standard lipid profile, HbA1c%, and liver function tests. A detailed medical history and prescription inventory were recorded and every participant underwent a standardised anthropometric assessment. After providing additional written informed consent, 202 volunteers with impaired glucose intolerance (IGT) or frank type 2 diabetes (defined by World Health Organisation (WHO) 1999 criteria) and no history of cardiovascular disease provided an additional blood and urine sample for the quantification of biomarkers of oxidative stress (2,3-dinor-8-iso-prostaglandin-F1*α*) and endothelial function (ICAM). 

All data and samples were obtained before any antihyperglycaemic medication dietary or lifestyle modifications were initiated. Informed consent was obtained from each participating subject, and the protocol was approved by the ethical committee of Leicestershire, Northamptonshire, and Rutland, UK.

### 2.2. Anthropometric Data

Anthropometric measurements were collected for each participant including height and weight (Tanita TBE 611, Tanita, West Drayton, UK), to the nearest 0.5 cm, 0.1 kg, and 0.5%, resp.). Waist circumference was measured at the point of minimal abdominal circumference located halfway between the navel and the lower end of the sternum [25]. Three separate blood pressure readings were taken (sitting without crossed legs at 5-minute intervals (Omron M5-1, HEM-757-E model) to the nearest 0.5 mmHg. The mean of the last two readings was used in these analyses.

### 2.3. Biochemical Data

Quantification of high-density lipoprotein (HDL) cholesterol was performed using the ultra-HDL assay (UHDL) and serum cholesterol using the cholesterol enzymatic assay. Serum triglyceride was measured using the triglyceride glycerol phosphate oxidase assay (Abbott Clinical Chemistry ARCHITECH c Systems/AEROSET systems). Quantification of serum glycohemoglobin (HbA1c%) was performed using high-performance liquid chromatography (HPLC) on the automated glycohemoglobin HLC-723G analyzer (Tosoh Bioscience Ltd., UK), and plasma glucose was measured using the Hexokinase method. These assays were undertaken in the pathology laboratories within University Hospitals Leicester and repeat testing carried out if the coefficient of variance was ≥20%.

### 2.4. 2,3-Dinor-8-iso-prostaglandin-F1*α* Assay

This analysis was conducted using an in-house assay on an AutoDELFIA 1235 automatic immunoassay system (PerkinElmer, Life Sciences, UK) at Unilever Colworth. All urine samples were collected and stored at −20°C until analyses took place. The quantification of human 2,3-dinor-8-iso-prostaglandin-F1*α* from urine was undertaken via a monoclonal antibody based competitive fluorescent immunoassay method with a 2,3-dinor-8-iso-prostaglandin-F1*α* (Cayman Chemical Company 15290) ovalbumin-Eu3^+^-labelled-tracer (EU N1 ITC chelate, PerkinElmer, Life Sciences, UK). Procedure: 50 *μ*L of the standard QC or urine sample were dispensed into a solid yellow low florescence anti-mouse 96-well microplate (PerkinElmer, Life Sciences, UK). The stock 2,3-dinor-8-iso-prostaglandin-F1*α* antibody (Unilever Discover) solution was diluted 1 : 100 in DELFIA assay buffer by the AutoDELFIA followed by the addition of 100 *μ*L of antibody (0.03 ug/mL final concentration) to each well of the plate. The stock Eu3^+^- labelled ovalbulin-8-Iso-prostaglandin-F1*α* tracer was automatically diluted 1/100 in assay buffer by the AutoDELFIA and then 50 *μ*L of this solution was added to each well (final dilution 1/16,000 tracer). A 60-minute incubation period followed with shaking and the plate was then washed 6 times with DELFIA wash buffer (PerkinElmer, Life Sciences, UK). 200 *μ*L of DELFIA enhancement solution (PerkinElmer, Life Sciences, UK) was then added to each well and the plate was shaken for 5 minutes. The fluorescence counts were then read from each well of the plate by the AutoDELFIA and the concentration of 2,3-dinor-8-iso-prostaglandin-F1*α* determined from the standard curve by the AutoDELFIA Multicalc data reduction programme. 

Repeat testing was carried out if the coefficient of precision was ≥10%. This assay utilises a monoclonal rather than polyclonal antibody as it affords greater specificity, and batch consistency. To evaluate the monoclonal antibody specificity an affinity chromatography matrix was prepared by linking 2,3-dinor-8-iso-prostaglandin-F1*α* monoclonal antibody to CNBr Sepharose 4B (Pharmacia). Column activity was demonstrated by the binding and elution of commercially available 2,3-dinor-8-iso-prostaglandin-F1*α* (Cayman Chemical Company). Good elution was obtained with 95% ethyl alcohol. Activity of the column following ethyl alcohol elution (ability to bind 2,3-dinor-8-iso-prostaglandin-F1*α*) was checked and found to be sufficient—indicating the column might be used for multiple purification runs. 14∗1 mL urine samples with 2,3-dinor-8-iso-prostaglandin-F1*α* immunoassay concentrations > 50 ng/mL were pooled and diluted in 28.5 mLs 0.1 M phosphate pH 7.1. The column was baselined with PBS and the diluted urine sample loaded at 1 mL/minute. The column was washed with PBS to baseline, then milli-Q water and then eluted with 5 mLs 95% ethyl alcohol followed by PBS. The elution peak, evaporated to dryness, was sent for analysis by GC-MS (Dr Erin Terry & Professor Jason Morrow, Vanderbilt University School of Medicine, Nashville, USA). The GC-MS tracings reported were clean, showing one clear single peak at about 0.20 minutes and later a smaller peak about 1/8 size of the sample peak. It was concluded the main peak was 2,3-dinor-8-iso-prostaglandin-F1*α* and the smaller peak an unknown metabolite. This result indicated high antibody specificity and contrasted with a previous result the Vanderbilt Laboratory had obtained with a commercially available monoclonal antibody, which showed multiple peaks, indicating poor antibody specificity. The DELFIA (PerkinElmer Life Sciences, UK) immunoassay format offers advantages over traditional ELISA immunoassay technology. It utilizes the unique chemical properties of lanthanide fluorescent chelates together with time-resolved fluorescence (TRF) detection to create an assay that offers high specificity, sensitivity, wide dynamic range, and superior stability. This methodology has been previously utilized by our group [26]. Urinary concentrations of this biological marker were then corrected by urinary creatinine concentration to account for differences in renal excretory function. 

### 2.5. ICAM Measurement

All serum samples were stood upright for 60 minutes at room temperature prior to centrifugation at 3000 rpm for 10 minutes. Each subsequent 2 mL aliquot was stored at −80°C until analyses took place. ICAM-1 was measured by ELISA (R&D systems, Abingdon, UK). The intra and inter-assay CVs were both <8%. 

### 2.6. Statistical Analysis

All statistical analyses were conducted using Stata 10.0 (StataCorp. 2007. *Stata Statistical Software: Release 10*. College Station, TX: StataCorp LP). Differences between South Asian and White European ethnic groups in terms of baseline characteristics, cholesterol, and glycaemia measures were assessed using *t*-tests for continuous variables and Fisher's exact tests for categorical variables. To compare oxidative stress, as measured by 2,3-dinor-8-iso prostaglandin-F1*α* between these two ethnic groups, an unadjusted linear regression model and a model adjusted for age (continuous; years), gender (categorical; male, female), smoking status (categorical; nonsmoker, exsmoker, current smoker), waist circumference (continuous; centimetres), HbA1c (continuous; %) and use of medication were fitted. 2,3-dinor-8-iso-prostaglandin-F1*α* was found to have a skewed distribution and so was log-transformed prior to analyses; back-transformed means are reported. Also of interest was whether oxidative stress was related to measures of glycaemia and the endothelial dysfunction marker ICAM. This was investigated using two different multiple regression models. The first model was an unadjusted model. The second model was adjusted for age, gender, ethnicity, smoking, medication use (including antihypertensive, lipid-lowering, thyroid/antithyroid medication, and steroids), and waist circumference. In all regression models, continuous explanatory variables were centred around their means (HbA1c = 6%, age = 58 years, waist circumference = 98 cm, fasting glucose = 6 mmol/L, 2-hour glucose = 9 mmol/L, ICAM-1 = 19 ng/mL). *P* values of 0.05 or lower were considered to be significant.

## 3. Results

Fifty-four per cent (109) of the cohort had WHO defined impaired glucose tolerance and 46% (93) had type 2 diabetes. The baseline characteristics are shown in Table 1. Thirty-four per cent (68) were of South Asian ethnicity and 66% (134) were of White European ethnicity. There was no significant difference in the prevalence of diabetes (*P* = 0.66), gender (*P* = 0.56), or waist circumference (*P* = 0.10) between the two ethnic groups; however, the white Europeans were significantly older compared to the south Asian group (62.7 years ±8.1 versus 54.9 years ±10.4, *P* < 0.001) and South Asians' had significantly lower body weight (75.9 ± 2.1 versus 87.4 ± 18.5, <0.001). 

Mean (±standard deviation) levels of LDL cholesterol and total cholesterol were significantly lower in the South Asian group compared to the White Europeans (LDL-C: 3.1 ± 0.8 versus 3.6 ± 1.0, *P* < 0.001, resp., and total cholesterol: 5.0 ± 1.0 versus 5.8 ± 1.2, *P* < 0.001, resp.). There were no differences between the two ethnic groups with respect to measures of glycaemia, as measured by fasting glucose (*P* = 0.10), 2-hour glucose (*P* = 0.35), and HbA1c% (*P* = 0.35). 

Mean unadjusted 2,3-dinor-8-iso-prostaglandin-F1*α* levels were significantly higher in the South Asian group compared with the White European group (mean 11.04 nmol/mmol_creatinine  _(95% CI: 9.85, 12.38) versus 9.03 nmol/mmol_creatinine  _ (95% CI: 8.32, 9.80), resp., *P* = 0.005). A similar difference was observed after adjustment for age, gender, smoking status, body weight, and HbA1c (Table 2). 

### 3.1. Standard Multiple-Regression 2,3-Dinor-8-iso-prostaglandin-F1*α* and Variables of Interest

A 1% increase in HbA1c was associated with a 1.09 nmol/mmol_creatinine  _ increase in oxidative stress (*β* =1.08, 95% CI: 1.02, 1.14, *P* = 0.009) after adjustment for age, gender, ethnicity, medication use, smoking status, and body weight in the whole cohort (Table 3). In this model levels of 2,3-dinor-8-iso-prostaglandin-F1*α* are 1.25 nmol/mmol_creatinine  _ (95% CI; 1.05, 1.49, *P* = 0.011, not in table) higher in South Asians than White Europeans independent of the potential confounding variables adjusted for. 

A 1 mmol/L increase in 2 hr glucose is associated with an average increase of 1.02 nmol/mmol_creatinine  _ in the level of oxidative stress *β* = 1.02, (95% CI: 1.00, 1.04, *P* = 0.042) adjusted for age, gender, ethnicity, medication use, smoking status and body weight in the whole cohort (Table 3). In this model levels of 2,3-dinor-8-iso-prostaglandin-F1*α* are 1.21 nmol/mmol_creatinine  _ (95% CI: 1.02, 1.43, *P* = 0.026, not in table) higher in South Asians than White Europeans independent of the potential confounding variables adjusted for. 

A 1 mmol/L increase in fasting blood glucose is associated with a 1.06 nmol/mmol_creatinine  _ increased level of oxidative stress (*β* = 1.06, (95% CI: 1.02, 1.10, *P* = 0.001) adjusted for age, gender, ethnicity, medication use, smoking status, and waist circumference in the whole cohort (Table 3). In this model the level of 2,3-dinor-8-iso-prostaglandin-F1*α* are 1.29 nmol/mmol_creatinine  _ (95% CI: 1.09, 1.54, *P* = 0.004, not in table) higher in South Asians than White Europeans independent of the potential confounding variables adjusted for. 

### 3.2. ICAM-1

For comparative purposes ICAM-1 was matched to a normal glucose tolerant control group. Mean values of ICAM-1 within this control population were significantly lower than in a composite impaired glucose tolerant and diabetes group among all subjects (*P* < 0.001, Table 3) and when South Asian (*P* = 0.015) and white European (*P* = 0.015) subjects were analysed separately. To further illustrate this association, logistic regression analyses were performed. We report for the combined cohort a 1 ng/mL increase in ICAM is associated with an 8% increased likelihood of IGR (OR 1.08 (95% CI: 1.03, 1.13) *P* < 0.001) which was similar for South Asians' and White Europeans' alone (OR: 1.09 (1.01, 1.17), *P* = 0.018 and 1.07 (1.01, 1.13), *P* = 0.015, resp.). No ethnic differences in ICAM-1 were observed in either normal glucose tolerant (*P* = 0.42) or impaired glucose regulation (*P* = 0.54) categories (Table 4). There was no statistically significant independent association between levels of ICAM and oxidative stress in this cohort (Table 4). 

## 4. Discussion 

In this study we demonstrate evidence of enhanced oxidative stress in apparently healthy first generation UK South Asians with undiagnosed hyperglycaemia. This difference of ~17% compared with an endogenous matched White European population is independent of measured confounding effects of age, gender, smoking, use of medication, body weight and HbA1c. Causality cannot of course be inferred from cross-sectional analyses of this nature. Nevertheless, our findings suggest that oxidative stress may contribute to accelerated atherogenesis and well-documented cardiovascular disease susceptibility within South Asians migrating to northern latitudes [1, 27–29]. 

Oxidative stress results from an imbalance between endogenous oxidant production and the level of antioxidant activity. It is established that a hypercalorific state (caloric intake exceeds energy expenditure) induces excess production of ROS via endoplasmic reticulum stress [30]. However, in a bid to reduce the formation of excess cellular ROS, and protect against its harmful effects, insulin stimulated glucose uptake is inhibited, via the reduction of GLUT-4 translocation, further exacerbating the hyperglycaemic state [31–34]. The pancreatic *β* cell is more susceptible to ROS due to a relative deficiency of antioxidant enzymes. Prolonged exposure to hyperglycaemia and free fatty acids in the hyper-caloric state is reported to induce *β*-cell dysfunction [32, 33]. This “glucolipotoxicity” has been described in a recent review [35] and oxidative stress identified as a major contributory factor to the process. Our study has demonstrated elevated levels of a marker of systemic oxidative stress in a UK South Asian cohort compared to White Europeans with comparable glycaemic status suggesting that South Asians could be susceptible to the detrimental effects of hyperglycaemia-induced oxidative stress at lower blood glucose thresholds. This is further supported by our multiple-regression analysis. Increasing levels of HbA1c, fasting, and 2-hour glucose were all associated with increasing levels of oxidative stress (Table 3). In each model, South Asians continued to have elevated levels of oxidative stress in comparison to White Europeans following adjustment for aforementioned confounders (Table 3). 

Conversely circulating cellular adhesion molecule values are not elevated in South Asians with either impaired glucose regulation or normo-glycaemia and no evidence of vascular disease compared to White Europeans (Table 4). This is perhaps not too surprising since whilst earlier studies suggested a strong link between ICAM-1 and vascular outcomes [22, 36–40], a later large prospective study and meta-analysis have contested this view [41]. Cell adhesion molecules including ICAM-1 were reported to have no significant relationship to CHD in their adjusted analysis. However, we report significantly higher levels of ICAM-1 in those with impaired glucose regulation compared to those with normo-glycaemia in the whole group and within ethnic groups. These results support recent findings from Sattar et al. [23] who reported a significant association between elevated ICAM-1 and risk of incident diabetes (HR 1.82 (95% CI: 1.27–2.63, *P* = 0.0013)). This group also reported no association between elevated ICAM-1 and incident CVD risk. This supports evidence from a smaller analysis of indigenous Indians demonstrating an upward progression of markers of lipid peroxidation and endothelial dysfunction (sVCAM-1) from healthy controls to diabetics with and without vascular disease [42]. Thus, our results support the growing body of evidence that dysregulated ICAM-1 is involved in the pathogenesis of T2DM but we suggest it is not attributable to the increased risk of vascular complications observed in South Asians residing in the UK. 

Potential limitations of the present study include its cross-sectional design. It is also observational and therefore subject to residual confounding. The F_2_ class of isoprostanes immerged as the “gold standard” assessment of in vivo oxidative stress and are the most widely documented of the isoprostane family [11–16, 43]. However, the F_2_-isoprostanes undergo further metabolism in the kidney yielding urinary dinor derivatives [43] including 2,3-dinor-8-iso-prostaglandin-F1*α*. Our group decided to quantify this urinary metabolite of oxidative stress in view of its relative stability and although our findings are unique in light of this we do not view our results to be of greater impact or novelty based on this methodology but consider them to be additive to the growing body of literature for the pathogenic role of oxidative stress in atherosclerosis and cardiovascular disease alike. However, because oxidative stress is a highly complex process with numerous unstable “midproducts” it is possible this urinary metabolite may not be sensitive enough to reflect the true extent of oxidative stress in this cohort or indeed at the tissue level. 

Although, only a single morning urine sample was used, we do not anticipate the alternative method of 24 hour sampling to have any influence on the results reported here. Helmersson and Basu have previously reported no significant variation between spot urine levels of F_2_-isoprostanes isomers and those measured from 24-hr urine sampling and in fact suggests that a morning sample is preferable as it is representative of urine from 6–8 hours [43, 44]. Isoprostane concentrations were however expressed/mmol creatinine to adjust for urine volume fluctuations and this urinary metabolite is a stable and validated marker of oxidative stress [44, 45]. A further limitation may be the use of surrogate subclinical markers of endothelial function. Although there is utility in the identification of endothelial phenotypes using circulating serum markers that are regulated and released by the vascular endothelium, flow mediated dilation (FMD) of the brachial artery is considered to be the gold standard technique for measuring endothelial function [46]. We therefore may have lost sensitivity by not employing this technique. Further, only one marker of endothelial dysfunction was used in this cohort and only a subset of participants provided the additional fasting blood for these analyses. This may have rendered it under powered and interpretation of secondary ICAM-1-isoprostane relationships should therefore be viewed with caution. Future investigation should include E-selectin and/or von Willebrand factor and a larger sample size. Furthermore, additional measures of oxidative stress, that is, 8-hydroxy-2′-deoxyguanosine (8-OHdG) may have substantiated these results further. 

Strengths of this study include the selection criteria which excluded confounders such as overt vascular disease and glucose-lowering treatments. Standard operating procedures were used throughout the study and laboratory analyses were blinded. The analysis of these biological markers was conducted using validated assay kits with good precision. Robust statistical techniques have been used which allowed us to include a larger number of potential confounders.

## 5. Summary

We show that ICAM-1 levels are not higher in South Asians residing in the UK despite its association with glycaemia in both groups. However, systemic oxidative stress appears to be significantly higher in glucose intolerant South Asians compared to their WE counterparts, independent of measured confounders. Our multiple-regression analysis show that HbA1c is positively associated with 2,3-dinor-8-iso-prostaglandin-F1*α* levels independent of confounding variables. The ethnicity variable in this model indicated that this positive association is greater in South Asians versus WE. The increase in 2,3-dinor-8-iso-prostaglandin-F1*α* was 1.09 nmol/mmol_creatinine  _ for the whole group for every 1% increase in HbA1c, for South Asians the increased levels of 2,3-dinor-8-iso-prostaglandin-F1*α* for every 1% increase in HbA1c was 1.25 nmol/mmol_creatinine  _ higher compared to WE. Subsequently, we suggest that South Asians are more susceptible to the detrimental effects of hyperglycaemia-induced oxidative stress at lower blood glucose thresholds than WE given that there is no significant difference in HbA1c between the ethnic groups in this cohort. To substantiate this potential relationship, further research into the mechanisms involved is warranted. 

## Figures and Tables

**Table 1:** Baseline characteristics.

Characteristic	*N* (%) or mean ± SD			*P* value for ethnicity^a^
	White Europeans	South Asians	Whole population
Total (*n*)	134	68	202	
Age, years	62.7 ± 8.1	54.9 ± 10.4	60.1 ± 9.7	<0.001
Gender				
Male	63 (47.0)	35 (51.5)	98 (48.5)	0.655
Female	71 (53.0)	33 (48.5)	104 (51.5)	0.556
Smoking status (current)	51 (38.1)	6 (8.8)	57 (28.2)	<0.001
Weight, kg	87.4 ± 18.5	75.9 ± 2.1	83.5 ± 18.5	<0.001
Waist circumference, cm	101.8 ± 15.0	98.0 ± 12.4	100.5 ± 14.3	0.096
Fasting glucose, mmol/L	6.8 ± 2.2	6.4 ± 1.4	6.7 ± 2.0	0.175
2-hour glucose, mmol/L	11.4 ± 4.2	11.3 ± 3.4	11.3 ± 3.9	0.809
HbA1c, *% *	6.6 ± 1.4	6.7 ± 0.9	6.6 ± 1.2	0.785
Type 2 diabetes	60 (44.8)	33 (48.5)	93 (46.0)	0.656
IGT	74 (55.2)	35 (51.5)	109 (54.0)	0.752
Blood pressure, mmHg				
Systolic	147.1 ± 17.9	141.0 ± 18.1	145.1 ± 18.2	0.041
Diastolic	88.8 ± 9.9	87.5 ± 10.2	88.4 ± 10.1	0.446
Cholesterol, mmol/L				
Total	5.8 ± 1.2	5.0 ± 1.0	5.5 ± 1.2	<0.001
LDL	3.6 ± 1.0	3.1 ± 0.8	3.4 ± 1.0	0.001
HDL	1.3 ± 0.3	1.2 ± 0.3	1.3 ± 0.3	0.010
Cardiovascular medication^b^	59 (42.4)	33 (48.5)	92 (45.5)	0.542

SD: standard deviation.

^a^ *P* values show the difference between the two ethnic groups and were calculated using *t*-tests for continuous variables and Fisher's exact test for categorical variables.

^b^Defined as any of following prescription medications: angiotensin converting enzyme inhibitor, beta-blocker, calcium channel antagonist, statin, or fibrate lipid lowering treatments.

**Table 2:** Mean 2,3-dinor-8-iso-prostaglandin-F1*α* in White European and South Asian ethnic groups.

	*N *	Mean^a^		*P* value^b^
		2,3-dinor-8-iso-prostaglandin-F1*α* nmol/mmol_creatinine_		
		(95% confidence interval)		
		White European	South Asian
Unadjusted	202	9.03 (8.32, 9.80)	11.04 (9.85, 12.38)	0.005
Adjusted^c^	172	8.46 (7.71, 9.29)	10.36 (9.09, 11.79)	0.021

^a^Estimated using linear regression on log-transformed values, and reported as back-transformed means.

^b^The *P* value showing the difference between the two ethnic groups was estimated using linear regression.

^c^Adjusted for age (continuous), gender (male, female), smoking status (non-smoker, ex-smoker, current smoker), weight (continuous), HbA1c (continuous) and medication use at point of data collection.

**Table 3:** Multiple regression analysis showing the effect of various glycaemia and endothelial dysfunction markers on 2,3-Dinor-8-Iso-prostaglandin-F1*α*.

Variable	Model	*R* ^2^	β**	95% CI	*P* value
HbA1c, %	Unadjusted	0.027	1.07	1.01, 1.13	0.019
	Adjusted for covariates	0.124	1.08	1.02, 1.14	0.009
2 hr Glucose, mmol/L	Unadjusted	0.016	1.02	1.00, 1.03	0.074
	Adjusted for covariates	0.112	1.02	1.00, 1.04	0.052
Fasting blood glucose, mmol/L	Unadjusted	0.028	1.04	1.01, 1.08	0.018
	Adjusted for covariates	0.141	1.06	1.02, 1.10	0.002
ICAM, ng/mL	Unadjusted	0.018	1.01	0.99, 1.03	0.183
	Adjusted for covariates	0.118	1.01	0.99, 1.03	0.297

*Adjusted for age, gender, ethnicity, medication use, smoking status, and weight. Only results for variable of interest and ethnicity are shown.

**Regression coefficients were estimated using log-transformed PGF and back-transformed values are reported.

**Table 4:** Mean ICAM-1 ng/mL within glucose categories of normal glucose tolerance (NGT) and impaired glucose regulation (IGR)* and stratified by ethnicity.

	Combined ICAM-1 population		South Asian		White European		*P* ^a^ (SA versus WE)
	*n *	Mean (95% CI)	*n *	Mean (95% CI)	*n *	Mean (95% CI)
NGT	141	17.30 (16.47, 18.14)	66	16.94 (15.70, 18.17)	75	17.62 (16.47, 18.77)	0.420
IGR*	183	19.51 (18.65, 20.38)	69	19.17 (17.87, 20.47)	114	19.72 (18.57, 20.88)	0.542
*P* ^a^ (NGT versus IGR)		<0.001		0.015		0.015	

CI: confidence interval, SA: South African, WE: White European.

IGR: Predefined composite of impaired glucose tolerance and type 2 diabetes.

^a^ *P* values were estimated using Student's *t*-tests.
